# Identification of a coherent twin relationship from high-resolution reciprocal-space maps

**DOI:** 10.1107/S2053273322002534

**Published:** 2022-04-28

**Authors:** Semën Gorfman, David Spirito, Guanjie Zhang, Carsten Detlefs, Nan Zhang

**Affiliations:** a Department of Materials Science and Engineering, Tel Aviv University, Wolfson Building for Mechanical Engineering, Tel Aviv, 6997801, Israel; b Electronic Materials Research Laboratory, Key Laboratory of the Ministry of Education and International Center for Dielectric Research, School of Electronic and Information Engineering, Xi’an Jiaotong University, Xi’an, People’s Republic of China; c European Synchrotron Radiation Facility, 72 avenue des Martyrs, Grenoble, 38043, France

**Keywords:** ferroelastic domains, domain walls, high-resolution X-ray diffraction

## Abstract

Twinning is a common crystallographic phenomenon which is related to the formation and coexistence of several orientation variants of the same crystal structure. It may occur during symmetry-lowering phase transitions or during the crystal growth itself. Once formed, twin domains play an important role in defining physical properties: for example, they underpin the giant piezoelectric effect in ferroelectrics, superelasticity in ferroelastics and the shape-memory effect in martensitic alloys. Regrettably, there is still a lack of experimental methods for the characterization of twin domain patterns. Here, a theoretical framework and algorithm are presented for the recognition of ferroelastic domains, as well as the identification of the coherent twin relationship using high-resolution reciprocal-space mapping of X-ray diffraction intensity around split Bragg peaks. Specifically, the geometrical theory of twinned ferroelastic crystals [Fousek & Janovec (1969). *J. Appl. Phys.* **40**, 135–142] is adapted for the analysis of the X-ray diffraction patterns. The necessary equations are derived and an algorithm is outlined for the calculation of the separation between the Bragg peaks, diffracted from possible coherent twin domains, connected to one another via a mismatch-free interface. It is demonstrated that such separation is always perpendicular to the planar interface between mechanically matched domains. For illustration purposes, the analysis is presented of the separation between the peaks diffracted from tetragonal and rhombohedral domains in the high-resolution reciprocal-space maps of BaTiO_3_ and PbZr_1−*x*_Ti_*x*_O_3_ crystals. The demonstrated method can be used to analyse the response of multi-domain patterns to external perturbations such as electric field, change of temperature or pressure.

## Abstract 2

The theory and algorithm are presented for the assignment of ferroelastic domains to the individual components of split Bragg peaks in high-resolution reciprocal-space maps. The formalism of mechanical compatibility of ferroelastic domains is further developed for the analysis of the geometry of the reciprocal space. The application of the algorithm to the reciprocal-space maps of tetragonal BaTiO_3_ and rhombohedral PbZr_1−*x*_Ti_*x*_O_3_ crystals is demonstrated.

## 1. Introduction

Twinning is a common crystallographic phenomenon (Cahn, 1954 ▸; Grimmer & Nespolo, 2006 ▸; Authier, 2003 ▸), related to the formation and coexistence of several orientation variants of the same crystal structure. The presence of twin domains may alter or even dominate material properties (Seidel, 2012 ▸; Catalan *et al.*, 2012 ▸; Tagantsev *et al.*, 2010 ▸), especially when a twin domain hosts order parameters of different physical nature (*e.g.* electric polarization and mechanical strain). Domain switching, domain rearrangement and domain-wall motion may underpin/enhance the technologically important piezoelectric effect (Hu *et al.*, 2020 ▸), dielectric permittivity (Damjanovic, 1998 ▸; Trolier-McKinstry *et al.*, 2018 ▸), superelasticity (Viehland & Salje, 2014 ▸), the shape-memory effect (Bhattacharya, 2003 ▸) and domain-wall superconductivity (Catalan *et al.*, 2012 ▸). The knowledge of domain patterns (*e.g.* average domain sizes and shapes, domain-wall orientations) is important for material design and properties engineering. Twinning is the subject of considerable interest in fundamental and applied science.

Lamentably, only a handful of experimental techniques are available for the experimental characterization of domain patterns (Wu *et al.*, 2015 ▸). These techniques are based on *e.g.* optical and birefringence microscopy (Ushakov *et al.*, 2019 ▸; Gorfman *et al.*, 2012 ▸), piezo response-force (PFM) (Gruverman *et al.*, 2019 ▸) microscopies or X-ray topography (Yamada *et al.*, 1966 ▸). Each of these said techniques contains some disadvantages, which limit the completeness and efficiency of the characterization. For example, optical microscopy is almost insensitive to the strain/lattice parameters, PFM is limited to the surface only. Accordingly, any new way of characterizing domains in the bulk would greatly contribute to the subject.

Single-crystal X-ray diffraction could potentially fill this methodological void. It is bulk penetrating, it is non-destructive and it has structural characterization power (*e.g.* sensitivity to the lattice parameters). Using synchrotron radiation adds the capabilities for *in situ* (*e.g.* stroboscopic) studies of domains at variable temperature and external electric field (see *e.g.* Zhang *et al.*, 2018 ▸; Gorfman *et al.*, 2020 ▸). The development of dark-field X-ray microscopy (Poulsen *et al.*, 2017 ▸; Kutsal *et al.*, 2019 ▸; Simons *et al.*, 2015 ▸) and coherent Bragg diffraction imaging methods (Robinson & Miao, 2004 ▸; Marçal *et al.*, 2020 ▸; Dzhigaev *et al.*, 2021 ▸) for combining reciprocal- and real-space information is another step towards advanced characterization of domain patterns. Nonetheless, despite the great potential of X-ray diffraction for the characterization of domain patterns, the technique remains far from routine (Harrison *et al.*, 2004 ▸). It is mainly because the distribution of X-ray diffraction intensity from a multi-domain crystal may be as complex as the domain patterns themselves. Up until now, single-crystal X-ray diffraction was successfully applied for characterization of domains in epitaxial thin films (Ehara *et al.*, 2017 ▸; Braun *et al.*, 2018 ▸; von Helden *et al.*, 2018 ▸; Lee *et al.*, 2019 ▸, 2020 ▸; Schmidbauer *et al.*, 2020 ▸) where possible domain patterns are greatly limited by the constraints imposed by the substrate.

We propose a framework for the recognition of a coherent twin relationship using high-resolution three-dimensional reciprocal-space mapping. Here the word ‘coherent’ describes the situation when two (or more) domains alternate and connect to one another without a lattice mismatch. If formed and stable, such twin domain patterns may significantly enhance the ability of a material to respond to external perturbations and thus enable new domain-related physical properties. Specifically, we focus on the assemblies of ferro­elastic domains (the volumes of a crystal where strain is uniform). While the method assumes the availability of the reciprocal-space information alone, it may also assist in the interpretation of the dark-field X-ray microscopy data.

The article has the following organization. After the introduction of the glossary of symbols and important relationships, we recapitulate the well known formalism for mechanical compatibility of coherent patterns of ferroelastic domains in a way that is suitable for the analysis of X-ray diffraction from them. Then we analyse the orientation relationship between their reciprocal lattices and calculate reciprocal-space separation of Bragg peaks of twinned ferroelectric domains. The demonstration of the method for the identification of a coherent twin relationship in domains of tetragonal (BaTiO_3_) and rhombohedral (PbZr_0.75_Ti_0.25_O_3_) symmetry is presented. We inspect diffraction from multi-domain ferroelectric crystals accordingly and display the means to assign different peak components to the individual domains.

## 2. Glossary of symbols and important relationships

We will consider how different ferroelastic domains may align with each other in a crystal. The calculation involves acquiring possible Miller indices of mismatch-free planes between domains, determination of the mutual orientation of one domain relative to another and obtaining reciprocal-space splitting of corresponding Bragg peaks. The goal of this section is to introduce central notations and relationships that assist in performing all the necessary real- and reciprocal-space operations in a concise manner.

**Figure 1:**
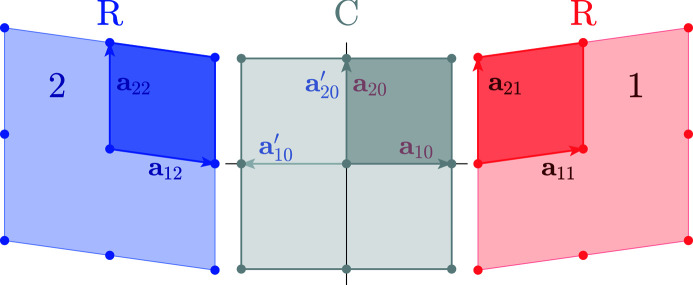
Schematic illustration of two-dimensional ferroelastic domains and their unit cells (2 × 2 supercells are shown). The middle image (marked by the letter C, standing for the two-dimensional prototype of ‘cubic’) corresponds to the single-domain ‘parent’ phase. The lattice basis vectors here are **a**_*i*0_ or, symmetry equivalently, 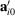 . The right and left images (marked by the letter R, standing for the two-dimensional prototype of ‘rhombohedral’) correspond to the ferroelastic domains. The lattice basis vectors here (**a**_*im*_, *m* = 1…2) are chosen in such a way that **a**_*im*_ are nearly parallel to **a**_*i*0_.

*Basis vectors*. **a**_*im*_ (*i* = 1…3) are the basis vectors of a crystal lattice. {The terms lattice and structure are often misused in the recent materials science literature [as noticed by Nespolo (2019 ▸)]. Therefore, we emphasize that ‘a crystal lattice’ refers to a regular array of points accounting for the periodicity of the structure. In contrast, ‘a crystal structure’ is obtained by translating a unit cell to all the points of a crystal lattice.} The second index refers to the ferroelastic domain variant *m*. *m* = 0 corresponds to the crystal lattice of a higher-symmetry (*e.g.* cubic) ‘parent’ phase (Fig. 1 ▸). The parallelepiped based on the vectors **a**_*im*_ forms a unit cell.

*Unit-cell settings*. Many unit-cell settings (choices of the basis vectors) exist for the same lattice (Gorfman, 2020 ▸). Here, we prefer the cell settings **a**_*im*_ (*m* > 0) obtained by the smallest possible distortion/rotation of the parent-phase basis vectors **a**_*i*0_. Fig. 1 ▸ shows a two-dimensional illustration of two ferro­elastic domains and the settings **a**_*i*1_, **a**_*i*2_ and **a**_*i*0_ for the domains 1, 2 and 0.

*Metric tensor/matrix of dot products*. *G*_*ij*_ = **a**_*i*_ **a**_*j*_ is the metric tensor (Giacovazzo, 1992 ▸; Hahn, 2005 ▸). The corresponding 3 × 3 matrix [*G*]_*m*_ is the matrix of dot products for the domain variant *m*. Their determinants are 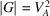 (*V*_*A*_ is the unit-cell volume). For a cubic lattice, 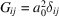 is valid (here *a*_0_ is the ‘cubic’ lattice parameter and δ_*ij*_ is the Kronecker symbol).

*The transformation matrix*. The transformation *e.g.* from the basis vectors **a**_*im*_ to the basis vectors **a**_*in*_ is defined by the 3 × 3 transformation matrix [*S*]. The columns of the matrix [*S*] are the coordinates of **a**_*in*_ with respect to **a**_*im*_:

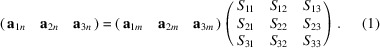




*Transformation of the metric tensor*. The transformation of the basis vectors (1) leads to the following transformation of the corresponding metric tensors:

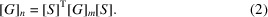

This relationship can be extended to any cases of transformation between coordinate systems.

*Twinning matrix*. [*T*] represents a symmetry operation of the parent-phase lattice (*i.e.* the one built using the basis vectors **a**_*i*0_) that is no longer the symmetry operation of a ferroelastic phase lattice. We define [*T*] as a 3 × 3 matrix, which describes the transformation to the coordinate system **a**_*i*0_ from its symmetry equivalent 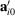 using the following formal matrix equation:

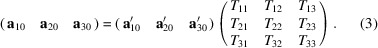

The number of symmetry-equivalent coordinate systems is equal to the order of the holohedry point symmetry group (*e.g.* 48 for a cubic lattice). The transition from a paraelastic to a ferroelastic phase is associated with the distortion of the basis vectors 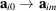 . Such distortion, however, can commence from any of the symmetry-equivalent 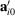 . Let us assume that **a**_*i*0_ and 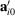 serve as the starting points for domain variants *m* and *n* correspondingly. Appendix *A* [App appa] demonstrates the proof that the following relationship between [*G*_*n*_] and [*G*_*m*_] exists:

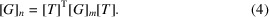

The two-dimensional example in Fig. 1 ▸ shows ferroelastic domains 1 and 2. The metric tensor of the domain 1 is

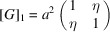

(here η is the cosine of the angle between the basis vectors). Domain 2 is related to domain 1 by twinning matrix

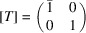

so that

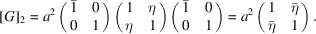




The following group of notations is introduced for the compactness of the description of the connectivity of the lattices of domains *n* and *m*:

[Δ*G*] = [*G*]_*n*_ − [*G*]_*m*_ is the difference between the metric tensors of the domains *n* and *m*. The reference to domain numbers in [Δ*G*] is omitted below.

*Eigenvalues and eigenvectors*. λ_1_, λ_2_, λ_3_ are the eigenvalues of [Δ*G*], [*V*] is the 3 × 3 matrix, whose columns are the corresponding eigenvectors. The condition Δ*G*_*ij*_ = Δ*G*_*ji*_ means that λ_*i*_ are real and that [*V*] is orthogonal ([*V*]^−1^ = [*V*]^T^).

*Supplementary coordinate systems*. The coordinate system **v**_*i*_ is defined by the matrix [*V*] so that

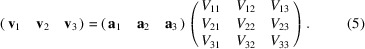

The coordinate systems 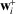 and 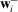 are introduced when λ^(1, 3)^ ≠ 0.:

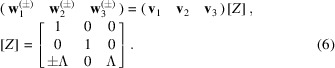

Here

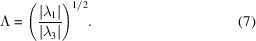

The transformation 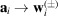 is given by the matrix [*W*] so that

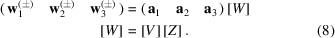

We will further omit the superscript (±) for brevity but keep in mind that **w**_*i*_ can be defined with *Z*_31_ = +Λ or *Z*_31_ = −Λ.

*Vector coordinates*. *x*_*i*_, *v*_*i*_, *w*_*i*_ are the coordinates of an arbitrary vector with respect to the coordinate systems **a**_*i*_, **v**_*i*_ and **w**_*i*_, so that *x*_*i*_ **a**_*i*_ = *v*_*i*_ **v**_*i*_ = *w*_*i*_ **w**_*i*_. The following direct and inverse transformations between the coordinates apply:

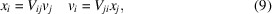

and

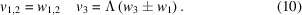




*Supplementary metric tensors*. [*G* ^(*V*)^], [*G* ^(*W*)^] are defined by the dot products 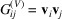 , 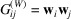 . They can be calculated analogously with (2) as [*G* ^(*V*)^] = [*V*]^T^[*G*][*V*] and [*G* ^(*W*)^] = [*W*]^T^[*G*][*W*].

*Reciprocal basis vectors*. The superscript * refers to the reciprocal bases, *e.g.* 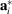 or 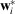 are such that 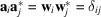 . The reciprocal metric tensor is 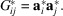


*Transformation between the reciprocal basis vectors*. If the direct basis vectors (*e.g.* **a**_*im*_ and **a**_*in*_) are related by the matrix [*S*] [according to equation (1)], then the corresponding reciprocal-lattice vectors ( 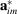 and 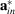 ) are related by the matrix [*S* ^*^]. The following relationship between [*S*] and [*S* ^*^] holds:

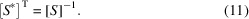




*Reciprocal coordinates of a vector*. 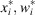 are the coordinates of an arbitrary vector with respect to the reciprocal coordinate system 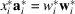 . The vector, indicating the position in the reciprocal space/lattice, is denoted by **B**. We also use the notations *hkl* for the indices of a plane and *HKL* for the indices of a Bragg reflection.

## 3. Mismatch-free connection of domains

This section recapitulates the approach of Fousek & Janovec (1969 ▸), Sapriel (1975 ▸) for the description of the geometrical connectivity of ferroelastic domains. Notably, it disregards the connectivity of atoms [*e.g.* oxygen octahedra in perovskites (Beanland, 2011 ▸)] but instead considers connectivity of lattices only. The lattices *n* and *m* are considered as connected if they meet along their common (*hkl*) plane so that those have exactly the same in-(*hkl*)-plane two-dimensional lattice parameters. The theory of martensitic phase transformations (Bhattacharya, 2003 ▸) refers to such planes as ‘habit planes’. All the points in this plane should have coordinates *x*_*i*_ such that

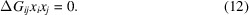

We are searching for the cases when (12) can be reduced to the equation of a plane:

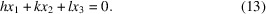

Let us transform the coordinates *x*_*i*_ to *v*_*i*_ according to (9) [and so the coordinate system **a**_*i*_ to **v**_*i*_ according to (5)]. Considering that the columns of [*V*] are the eigenvectors of [Δ*G*] simplifies (12) to

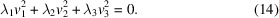

Equation (14) can be rewritten as (13) if one of the eigen­values is zero (*e.g.* λ_2_ = 0). Two cases may be considered:

The case λ_1_ = λ_2_ = 0, λ_3_ ≠ 0 yields

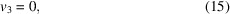

and represents the (001)_*v*_ plane (the subscript *v* refers to the Miller indices with respect to **v**_*i*_ instead of **a**_*i*_). Using (9), we reformulate (15) as

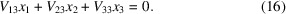

Extending the components *V*_13_, *V*_23_, *V*_33_ to the integer numbers will give the Miller indices *hkl* of the mismatch-free plane.

The case λ_1_ < 0, λ_2_ = 0, λ_3_ > 0 leads to two possible plane solutions of (14):

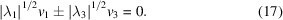

Using (9) and (7) can help to rewrite (17) as

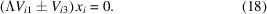

Extending the components of (Λ*V*_*i*1_ ± *V*_*i*3_) to the integer numbers would give the Miller indices *hkl* of the habit planes between the domains. Alternatively, the indices of the habit plane can be expressed using the coordinate system **w**_*i*_ correspondingly. Indeed, substituting (10) into (18) will give

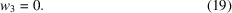

Accordingly, (19) can be described as (001)_*w*_ (the subscript *w* refers to the Miller indices with respect to the coordinate system **w**_*i*_ instead of **a**_*i*_).

This formalism gives a well known result for the Miller indices of the possible habit planes between the domains of different symmetry. Some examples are presented further in Sections 6 and 7.

## 4. Mutual orientation of domains

The goal of this section is to find the transformation matrix [*S*] [as defined by (1)] between the basis vectors **a**_*im*_ and **a**_*in*_ when the lattices of the domains *m* and *n* meet along their common (001)_*w*_ plane. Let us first find the similar transformation matrix [*S*_*w*_] between **w**_*im*_ and **w**_*in*_. The matching along the (001)_*w*_ plane implies **w**_1,2*m*_ = **w**_1,2*n*_ so that

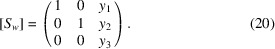

The unknown coefficients *y*_*i*_ can be calculated as follows. First, consider that the determinant of the transformation matrix (|*S*_*w*_| = *y*_3_) should be equal to the ratio of the unit-cell volumes; therefore

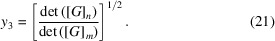




Note that, although *y*_3_ = 1 for the case when domains have the same unit-cell volume, the formalism is valid when matching between the lattices of different phases is in question (*y*_3_ ≠ 1). *y*_1_, *y*_2_ can be found substituting (2) ([*G* ^(*W*)^]_*n*_ = [*S*_*w*_]^T^[*G* ^(*W*)^]_*m*_[*S*_*w*_]) and (20) into

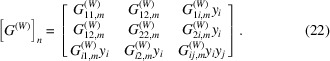

Comparing the elements 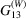 and 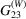 of the matrices, we get

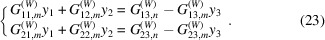

Solving this system of linear equations gives the values of the remaining coefficients *y*_1_, *y*_2_ and accordingly all the elements of the matrix  [*S*_*w*_]. Finally, the [*S*] can be found according to the equation

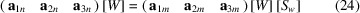

which immediately leads to

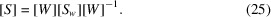




Accordingly, the elements of the matrix [*S*] can be found by going through the following steps.

(i) Choosing appropriate twinning matrices and calculating the elements of the corresponding metric tensors [*G*]_*m*_ and [*G*]_*n*_ using (4).

(ii) Calculating the eigenvectors ([*V*]) and eigenvalues (λ_*i*_) of [Δ*G*] = [*G*]_*n*_ − [*G*]_*m*_. Mismatch-free connection of domains *m* and *n* is possible only if at least one eigenvalue is zero, while the remaining two have opposite signs.

(iii) Rearranging the eigenvalues and eigenvectors in such a way that λ_1_ ≤ 0, λ_2_ = 0, λ_3_ > 0.

(iv) Using these eigenvalues and eigenvectors to form two pairs of matrices [*Z*] and [*W*] (either with *Z*_31_ = Λ or *Z*_31_ = −Λ) according to equations (5), (6), (7), (8). For the cases when two eigenvalues of [Δ*G*] are zero, [*Z*] is a unitary matrix.

(v) Calculating [*G* ^(*W*)^]_*m*_ and [*G* ^(*W*)^]_*n*_ according to equation (2) and determining the coefficients *y*_1_, *y*_2_, *y*_3_ using (21) and (23). Setting the matrix [*S*_*w*_] according to (20) and converting it to [*S*] according to (25).

The corresponding numerical examples will be presented in Sections 6 and 7.

## 5. Separation between the Bragg peaks

**Figure 2:**
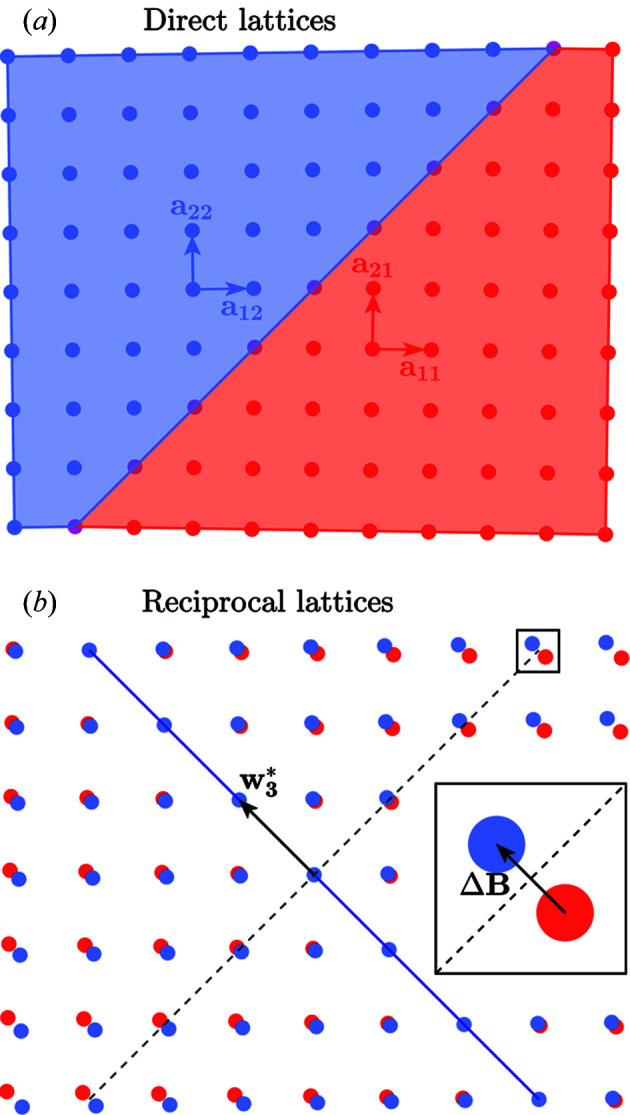
Two-dimensional illustration of direct and reciprocal lattices of two domains. (*a*) The lattices of two two-dimensional tetragonal (= rectangular) domains connected along their common 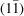 plane. (*b*) Their reciprocal lattices. The dashed line is parallel to the 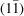 plane (domain wall); the inset highlights the separation between corresponding reciprocal-lattice vectors, showing that it is perpendicular to the domain wall.

Different twin domains would diffract X-rays into slightly different directions. The corresponding nodes of the reciprocal lattices can almost overlap in some cases but be fully resolved in others. The examples of real diffraction patterns of perovskite-based crystals with ferroelastic domains can be seen in the work of Gorfman & Thomas (2010 ▸), Gorfman *et al.* (2011 ▸, 2020 ▸), Choe *et al.* (2018 ▸), Zhang *et al.* (2018 ▸). Fig. 2 ▸(*a*) shows a two-dimensional example of domains, matching along ( 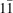 ) planes. Fig. 2 ▸(*b*) shows their reciprocal lattices. The goal of this section is to calculate the separation Δ**B** between the Bragg peaks *H* *K* *L* of two matched domain variants *m* and *n*. Specifically, we will derive the coordinates Δ*H*Δ*K*Δ*L* of Δ**B** relative to the reciprocal basis vectors of the domain *m* ( 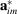 ). Let us first calculate the 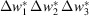 coordinates of Δ**B** relative to 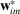 . We express Δ**B** in the form

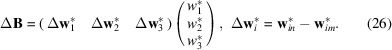




Considering that 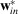 and 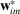 are related by the matrix 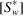 we get

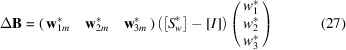

so that

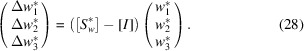




Using (20) and considering that 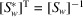 we get

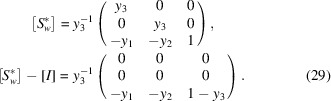

Here [*I*] is the unitary matrix.

Substituting (29) into (28) we obtain

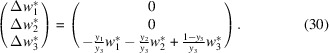

This also means that

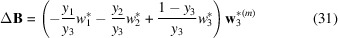

so that Δ**B** ∥ 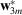 . Considering that 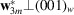 , we conclude that Δ**B** is normal to the domain wall. This statement is graphically illustrated in Fig. 2 ▸(*b*), which shows two lattices matched along the 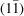 plane.

Note that expression (28) can be reformulated in order to express the separation vector relative to the coordinate system 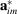 :

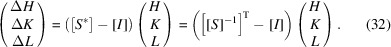




The next two sections demonstrate the formalism on the examples of domains of tetragonal and rhombohedral symmetry.

## 6. Examples

### 6.1. Tetragonal domains

**Figure 3:**
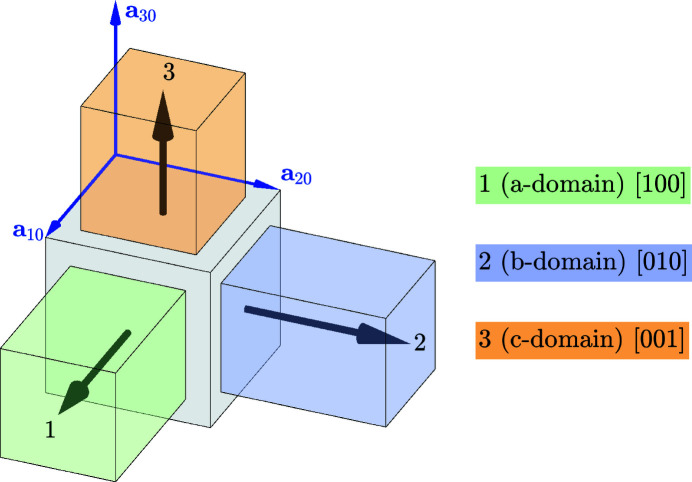
Definition and numbering of the tetragonal domain variants. The direction of the unique (fourfold symmetry) axis with respect to the basis vectors of the domains **a**_*im*_ (*m* = 1…3) is given. It is [100] for the *a* domain, [010] for the *b* domain and [001] for the *c* domain. This is also the direction of the spontaneous polarization in the case when the structure is polar.

Let us assume that the lattices of the paraelastic/ferroelastic phases belong to the cubic/tetragonal point symmetry groups *m*3*m* / 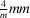 . Because these groups contain 48 and 16 symmetry operations, respectively, the phase transition between them results in the formation of three domain variants (Fig. 3 ▸ and Table 1 ▸). The ‘naming’ of the domains (*a* domain, *b* domain, *c* domain) reflects the direction of the unique axis (fourfold symmetry axis in this case). For the case when the actual crystal structure is polar, this axis coincides with the direction of a spontaneous polarization.

Consider the connectivity between the domains 1(*a*) and 3(*c*). Following Section 3 we get

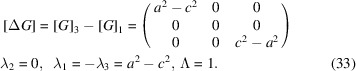

Because [Δ*G*] is diagonal and λ_2_ = 0 we can set [*V*] = [*I*] and immediately obtain that [according to equation (18)] domains may match along 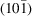 or (101) planes.

For the case of the 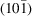 domain wall:

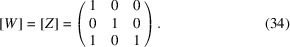




To formulate the system of equations (23) we need to calculate the matrices [*G* ^(*W*)^]_1, 3_ = [*W*]^T^[*G*]_1, 3_[*W*]. Using (34) and Table 1 ▸ we get

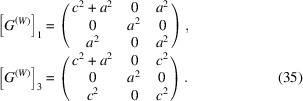




We now have to find the unknown coefficients *y*_1_, *y*_2_, *y*_3_. According to (21) *y*_3_ = 1. The system of equations (23) can be rewritten as

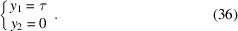

Here we introduced the notation

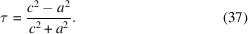

Using (36) and (20) we obtain

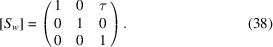

Now we substitute (34) and (38) into (25) and get the following equations for [*S*] and [*S* ^*^]:

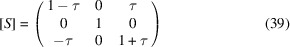

and using equation (11)


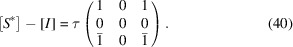

This finally gives the following expression for the separation between *HKL* Bragg peaks, diffracted from the domains 1(*a*) and 3(*c*):

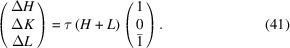




An identical analysis can be implemented for the (101) domain wall and other pairs of domains. Table 2 ▸ summarizes the results: it includes all the possible mismatch-free domain walls and corresponding coordinates of Δ**B** in the reciprocal coordinate system of the domain *m*. Note that the separation vector Δ**B** is always perpendicular to the domain wall.

### 6.2. Rhombohedral (trigonal) domains

**Figure 4:**
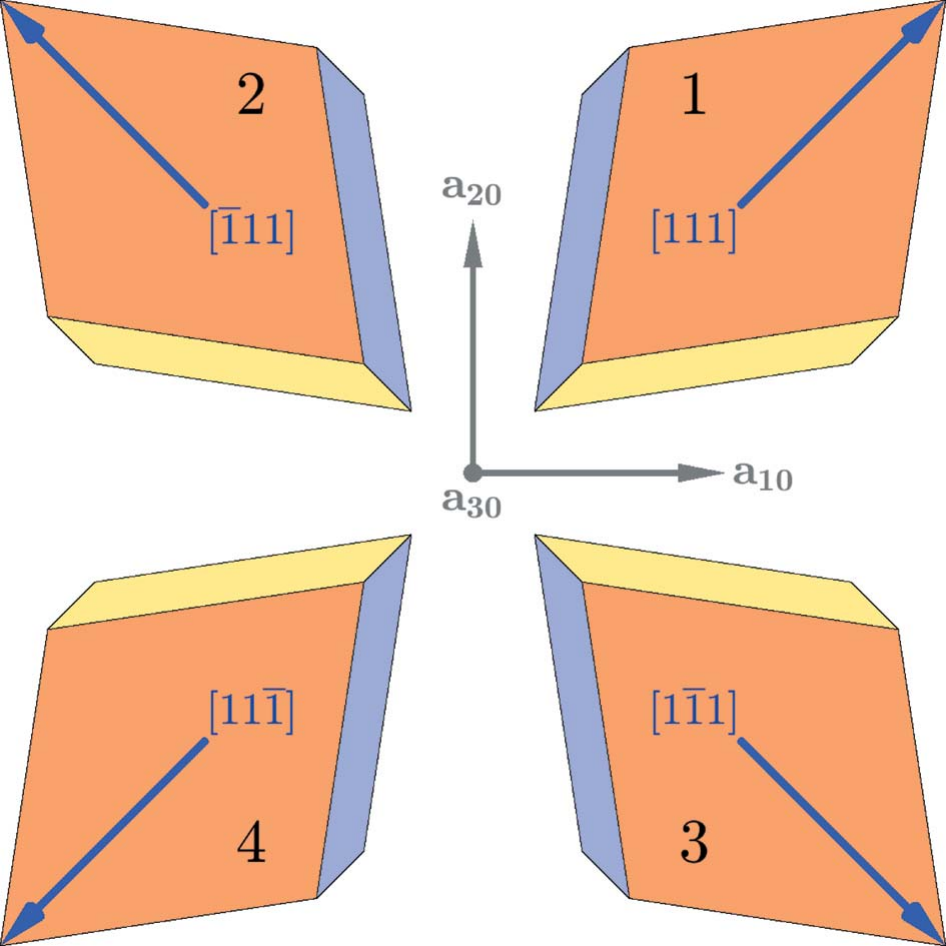
The definition and numbering of four rhombohedral domain variants. The direction of the unique axis (threefold symmetry axis in this case) is given relative to the basis vectors **a**_*im*_. These directions coincide with the direction of the spontaneous polarization for the case where the structure is polar. The basis vectors of the paraelastic phase **a**_*i*0_ are shown in the figure.

Let us assume that the lattices of the paraelastic/ferroelastic phases belong to the cubic/trigonal point symmetry group *m*3*m* / 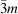 , containing 48 and 12 symmetry operations correspondingly. Accordingly, an 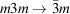 transition results in the formation of four domain variants. These domains are illustrated in Fig. 4 ▸ and Table 3 ▸. Analogically to the case of tetragonal domains, we will also identify these domains by the direction of the unique symmetry axes (in this case threefold symmetry axis) parallel to [111], 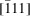 , 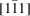 , 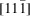 correspondingly.

Six pairs may be formed between four ferroelastic domain variants. We will demonstrate the connection between domains 1 and 2:

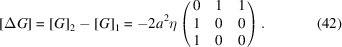

The eigenvalues and eigenvectors of (42) are such that

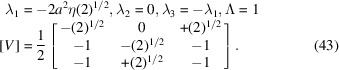




Equation (18) (*V*_*i*1_ ± *V*_*i*3_)*x*_*i*_ = 0 takes the form

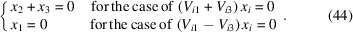

The first part of equation (44) corresponds to the (011) plane, while the second part corresponds to the (100) one. This is a well known result (Fousek & Janovec, 1969 ▸), indicating that rhombohedral domains may pair along the families of domain walls with the Miller indices {011} and {100}.

For the case of (011) domain walls, the transformation matrix [*W*] = [*V*][*Z*] will have to be introduced with

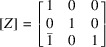

so that

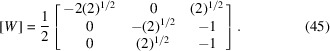

To formulate the system of equations (23) we will first get [*G* ^(*W*)^]_1, 2_ = [*W*]^T^[*G*]_1, 2_[*W*]:

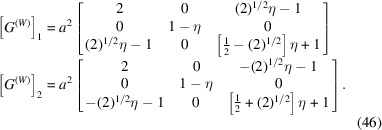




According to (21), *y*_3_ = 1 and applying the system of equations (23) we get

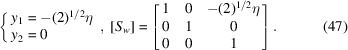

According to (25) and (45) we get for the matrix [*S*] = [*W*][*S*_*w*_][*W*]^−1^:

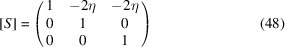

and

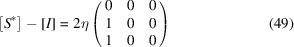

which yields the following expression for the separation between the Bragg peaks:

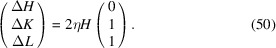




For the case of the (100) domain wall, the transformation matrix [*W*] = [*V*][*Z*] will have to be introduced with

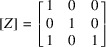

so that

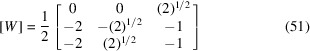

so that

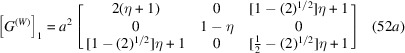




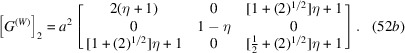




According to (21), *y*_3_ = 1 and applying equation (23) we get

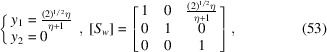

and according to equations (25) and (45) we can get for the matrix [*S*]:

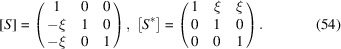




Here the following notation was introduced:

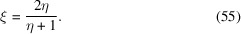

Accordingly

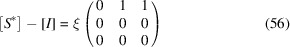

which finally gives the following expression for the separation between peaks:

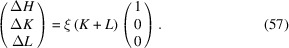

An identical analysis can be implemented to the domain wall and other pairs of domains. Table 4 ▸ summarizes the results: it includes all the possible domain walls between possible domain variants *m* and *n* and corresponding separation of Bragg reflections in the reciprocal coordinate system of the domain *m*. As can be seen, the separation vector is always perpendicular to the domain wall.

## 7. Experimental method: three-dimensional high-resolution reciprocal-space mapping

The experimental details of high-resolution reciprocal-space mapping were explained elsewhere (Gorfman *et al.*, 2020 ▸; Zhang *et al.*, 2018 ▸). The technique uses a parallel and monochromatic X-ray beam alongside a high-resolution pixel area detector. The goal of the experiment is to reconstruct the fine details of the diffraction intensity distribution around specific Bragg peaks. It allows one to measure the separations of nearly overlapping Bragg peak components (each corresponding to a separate domain). The intensity distribution in the reciprocal space is reconstructed by rotating the crystal around one of the diffractometer axes (*e.g.* ω) and converting three coordinates *X*_d_ *Y*_d_ ω (*X*_d_ *Y*_d_ are the coordinates of the detector pixels) to the coordinates of the scattering vector **B** relative to the chosen Cartesian (*B*_*x*_, *B*_*y*_, *B*_*z*_) coordinate system. Such experiments are facilitated by the recent progress in synchrotron-based and home-laboratory X-ray sources, availability of pixel area detectors, beam conditioning systems and big-data exchange protocols (see *e.g.* Dyadkin *et al.*, 2016 ▸; Girard *et al.*, 2019 ▸; Gorfman *et al.*, 2021 ▸).

## 8. Recognition of a coherent twin relationship in a tetragonal BaTiO_3_ crystal

**Figure 5:**
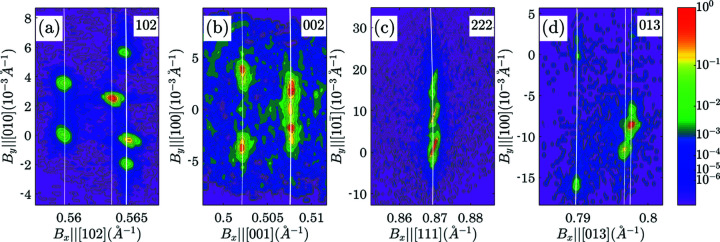
*I*_*z*_(*B*_*x*_, *B*_*y*_) projections of the reciprocal-space maps of 102, 002, 222 and 013 reflections from a BaTiO_3_ crystal containing a ferroelastic domain of tetragonal symmetry. The white lines correspond to the equation 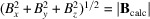 [here |**B**_calc_| was calculated using tetragonal lattice parameters *a* = 3.962 (1), *c* = 4.005 (2) Å].

This section illustrates the recognition of a coherent twin relationship in a twinned BaTiO_3_ crystal. We performed high-resolution reciprocal-space mapping measurements at the dedicated home-laboratory X-ray diffractometer at Tel Aviv University (Gorfman *et al.*, 2021 ▸). Following the determination of the average orientation matrix using *CrysAlisPro* software (the averaging is performed over all the domains present in the X-ray beam), we collected high-resolution reciprocal-space maps of the diffraction intensity distribution around 102, 002, 222 and 103 reflections. The data were then represented in the form of three-dimensional diffraction intensity tables *I*(*B*_*x*_, *B*_*y*_, *B*_*z*_). Here *B*_*x*_, *B*_*y*_, *B*_*z*_ refer to the Cartesian coordinate system such that the *X* axis is nearly parallel to the scattering vector, so that *B*_*x*_ is almost equal to the scattering vector length. Fig. 5 ▸ shows *B*_*x*_, *B*_*y*_ projections 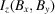 = 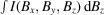 . Such projections visually demonstrate the separation of sub-peaks along the *X* axis (nearly equivalent to the separation along the 2θ axis). This splitting of the peaks along the *X* axis can be used to determine tetragonality or (in the more general case) the deviation of the lattice parameters from that of the cubic system.

The initial assignment of the sub-peaks to domains can be solely based on the analysis of the scattering vector lengths. The procedure was described by Gorfman *et al.* (2020 ▸). It includes measuring the lengths of the scattering vector of all the observed sub-peaks using the equation 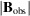 = 
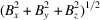
 . These are matched with the calculated lengths of the reciprocal-lattice vectors 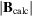 = 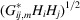 (*H*_1_ = *H*, *H*_2_ = *K*, *H*_3_ = *L* are the indices of the reflection). The lattice parameters are adjusted to achieve the optimal match between the sets of |**B**_obs_| and |**B**_calc_|. Using combined analysis of the scattering vector lengths in four reciprocal-space maps, we obtained tetragonal lattice parameters *a* = 3.962 (1), *c* = 4.005 (2) Å. According to (37) τ = 0.011 (1). The white lines in the *I*_*z*_(*B*_*x*_, *B*_*y*_) projections in Fig. 5 ▸ follow the equation 
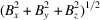
 = 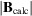 , where *B*_*z*_ corresponds to the *Z* coordinate of the best matching sub-peak.

**Figure 6:**
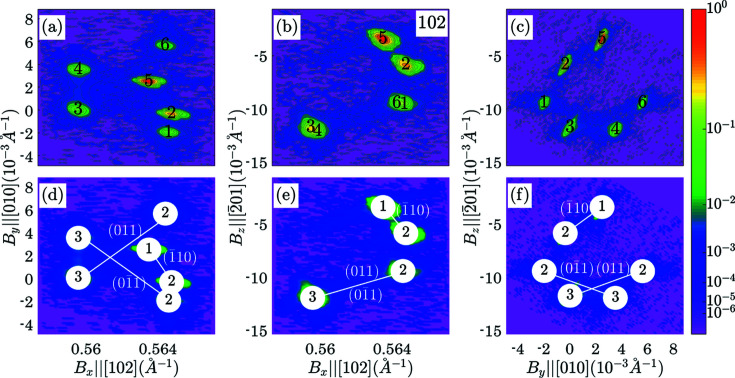
*I*_*z*_(*B*_*x*_ *B*_*y*_), *I*_*y*_(*B*_*x*_ *B*_*z*_) and *I*_*x*_(*B*_*y*_ *B*_*z*_) projections of three-dimensional diffraction intensity distribution *I*(*B*_*x*_, *B*_*y*_, *B*_*z*_) around the 102 family of Bragg peaks of BaTiO_3_. The panels (*a*)–(*c*) show six sub-peaks that are located and numbered in the maps. The panels (*d*)–(*f*) show the assignment of the peaks to the domains (as presented in Tables 5 ▸ and 6 ▸). The solid lines connect the peak pairs, which correspond to the matched domains. The Miller indices of the matching plane are indicated in the brackets.

The example below demonstrates the assignment of peaks to domains and identification of a coherent twin relationship in the corresponding reciprocal-space maps. Fig. 6 ▸ presents the corresponding *I*_*z*_(*B*_*x*_,*B*_*y*_), *I*_*y*_(*B*_*x*_,*B*_*z*_) and *I*_*x*_(*B*_*y*_,*B*_*z*_) projections. Figs. 6 ▸(*a*)–6 ▸(*c*) show the marked positions of the peaks [according to the procedure of Gorfman *et al.* (2020 ▸)]. Table 5 ▸ summarizes the results of this marking. For each marked peak, it includes the observed and calculated length of the reciprocal-lattice vector [*e.g.* 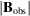 = 
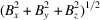
 and 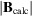 = 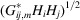 ] as well as one possible assignment of peaks to the domains [the domain number(s) *m* for which the best matching between |**B**_obs_| and |**B**_calc_| is achieved]. Finally, the last three rows of Table 5 ▸ illustrate the reciprocal-lattice coordinates of the peaks with respect to the mass centre. A similar analysis of three other reciprocal-space maps is presented in the supporting information.

Figs. 6 ▸(*d*)–6 ▸(*f*) illustrate the result of the identification of the coherent twin relationship. They show the same projections of three-dimensional intensity distribution with the relevant connections between the sub-peaks. The sub-peaks are connected with each other if the separation between them (the fifth column of Table 6 ▸) matches one of the theoretically predicted values (the fourth column of Table 6 ▸).

According to Table 6 ▸ and Figs. 6 ▸(*d*)–6 ▸(*f*), all three tetragonal domains are present in the relevant volume of the crystal (exposed by the X-ray beam during the collection of this reciprocal-space map). The following coherent twin relationship among them can be identified:

(i) 1(*a*) and 2(*b*) domains, connected to each other via the 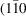 ) domain wall.

(ii) 2(*b*) and 3(*c*) domains, connected to each other via the 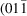 ) domain wall.

(iii) 2(*b*) and 3(*c*) domains, connected to each other via the (011) domain wall.

## 9. Recognition of ferroelastic domains in a PbZr_0.75_Ti_0.25_O_3_ rhombohedral single crystal

**Figure 7:**
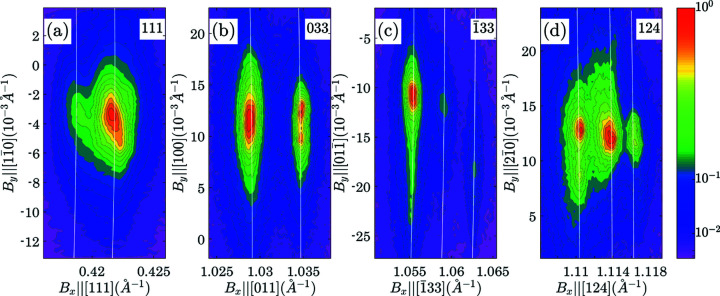
Same as Fig. 5 ▸ except for the reciprocal-space maps of 111, 033, 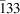 and 124 reflections from a twinned PbZr_0.75_Ti_0.25_O_3_ crystal containing domains of rhombohedral symmetry. The white lines correspond to the reciprocal-lattice vector lengths, which are calculated using rhombohedral lattice parameters *a* = *b* = *c* = 4.115 (1) Å, α = β = γ = 89.686°.

This section illustrates the recognition of a coherent twin relationship in the high-resolution X-ray diffraction patterns of a twinned PbZr_0.75_Ti_0.25_O_3_ crystal. The data were collected exactly as in the case of the BaTiO_3_ crystal (Section 8) and at the custom-built diffractometer at Tel Aviv University. Fig. 7 ▸ (organized as Fig. 5 ▸) shows *I*_*z*_(*B*_*x*_, *B*_*y*_) projections of diffraction intensity distribution. Similar to the case of BaTiO_3_ the separation of the peaks along the *X* axis can be used to determine the rhombohedral distortion. The rhombohedral lattice parameters *a* = 4.115 (1) Å, γ = 89.686° were obtained. Accordingly η = 0.0055 and ξ = 0.0109.

**Figure 8:**
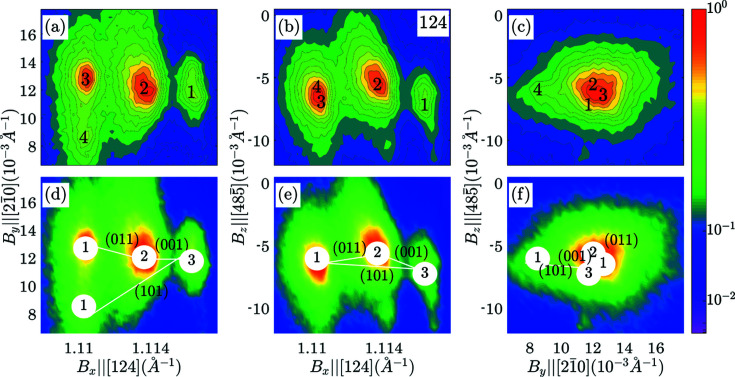
*I*_*z*_(*B*_*x*_ *B*_*y*_), *I*_*y*_(*B*_*x*_ *B*_*z*_) and *I*_*x*_(*B*_*y*_ *B*_*z*_) projections of three-dimensional diffraction intensity distribution *I*(*B*_*x*_, *B*_*y*_, *B*_*z*_) around the 124 family of Bragg peaks of PZT (PbZr_0.75_Ti_0.25_O_3_). The panels (*a*)–(*c*) show four sub-peaks that are located and numbered in the maps. The panels (*d*)–(*f*) show the assignment of the peaks to the domains (as presented in Tables 7 ▸ and 8 ▸). The solid lines connect the peak pairs, which correspond to the matched domains. The Miller indices of the matching plane are indicated in the brackets.

The example demonstrates the assignment of the peaks in the reciprocal-space maps of the 124 reflection. Fig. 8 ▸ and Table 7 ▸ are organized in the same way as Fig. 6 ▸ and Table 6 ▸.

According to Table 8 ▸ and Fig. 8 ▸, the following coherent twin relationship between rhombohedral domains can be identified:

(i) 1 and 2 domains, connected via the (011) domain wall.

(ii) 2 and 3 domains, connected via the (001) domain wall.

(iii) 1 and 3 domains, connected via the (101) domain wall.

## 10. Discussion

The presented algorithm may be useful in many cases, *e.g.* for the investigation of the response of a multi-domain system to an external perturbation (*e.g.* temperature or electric field). Considering that the integrated intensities of the peaks are proportional to the volume fraction of the corresponding domains in the beam, it is possible to describe the evolution of the domain pattern quantitatively. This method was used for the estimation of extrinsic and intrinsic contributions to the electromechanical coupling in the PbZr_1−*x*_Ti_*x*_O_3_ single crystal (Gorfman *et al.*, 2020 ▸). The ability to assign peaks to domains allows one to relate the corresponding change in the domain volume fraction as a function of the domain orientation, including *e.g.* the direction of the spontaneous polarization vector with respect to applied perturbation (*e.g.* electric field).

We have demonstrated the procedure of domain recognition for the cases of crystals with tetragonal and rhombohedral domains. The same algorithm can be applied *e.g.* to the domains of other symmetry [*e.g.* monoclinic symmetry, as will be demonstrated in the upcoming publication(s)]. Moreover, it may also be used for the analysis of the connections of domains of different symmetry. Formation of the habit planes between domains is possible every time when at least one eigenvector of the matrix [Δ*G*] is zero. The implication of this condition in the cases when pairing of different phases (*e.g.* rhombohedral and tetragonal) is in question will also be discussed in the forthcoming publications.

It is vital that the presented technique will be able to recognize domain pairs, as opposed to the individual domains themselves individually. Accordingly, some of the peaks may remain unrecognized. Such cases are apparent *e.g.* in the 002 reciprocal-space maps (see Fig. S1 in the supporting information). Unpaired peaks may appear when *e.g.* the limited volume of the crystal is covered by an X-ray beam (because of strong absorption of an X-ray beam hiding some domains and keeping some of the peaks unpaired). This is the reason why assignment of peaks may fail in such cases. Note that an assignment may still be attempted based on the length of the reciprocal-lattice vector and the radial position of the peak in the reciprocal space. In some cases this means that suggestions of more than one domain for a single sub-peak may be possible.

## 11. Conclusions

We developed the theoretical framework for the calculation of three-dimensional splitting of Bragg peaks, diffracted from a crystal with ferroelastic domains. Specifically, we extended the existing theory of domains’ mechanical compatibility to calculate the corresponding geometry of the reciprocal space. We have shown (analytically) that Bragg peaks always separate along the reciprocal-space direction that is perpendicular to the domain wall. The analytical expression for the Bragg peak separation for the cases of the entire domain wall between domains of tetragonal and rhombohedral symmetry was obtained. The formalism is illustrated using the example of single-crystal X-ray diffraction from a multi-domain BaTiO_3_ crystal with tetragonal domains and a multi-domain PbZr_0.75_Ti_0.25_O_3_ crystal with rhombohedral domains. It can be useful for the analysis of the individual domains’ response to external perturbation (*e.g.* the change of temperature or external electric field).

## Supplementary Material

Supporting figures and tables. DOI: 10.1107/S2053273322002534/lu5017sup1.pdf

## Figures and Tables

**Table 1:** The definition of the tetragonal domain variants (Fig. 3 ▸) The first row assigns the number (name) to the domain variant; the second row shows the matrix of dot products [*G*]_*m*_; the third row shows the crystallographic direction of the unique axis with respect to the lattice basis vectors (**a**_*im*_).

Domain No. (name)	1 (*a*)	2 (*b*)	3 (*c*)
Metric tensor [*G*]_*m*_	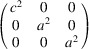	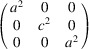	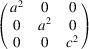
Direction of the unique axis	[100]	[010]	[001]

**Table 2:** A summary of possible mismatch-free domain walls between tetragonal domains and the corresponding separation between their Bragg peaks The first two columns show the domain numbers (names) *m* and *n* (according to Table 1 ▸ and Fig. 3 ▸). The third column shows the Miller indices of the mismatch-free plane. The fourth column shows the matrix [*S*] (the columns of this matrix are the coordinates of the vectors **a**_*in*_ relative to the domain  **a**_*im*_). The fifth column shows the matrix [*S* ^*^] − [*I*]. The last column shows the coordinates of the vector Δ**B** = **B**_*n*_ − **B**_*m*_ relative to the reciprocal basis vectors 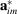 .

*m*	*n*	Plane	[*S*]	[*S* ^*^] − [*I*]	Δ**B**
1 (*a*)	2 (*b*)	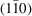	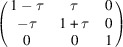	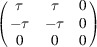	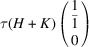
1 (*a*)	2 (*b*)	(110)	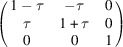	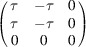	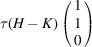
1 (*a*)	3 (*c*)	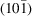	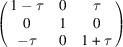	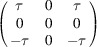	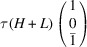
1 (*a*)	3 (*c*)	(101)	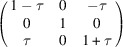	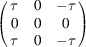	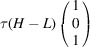
2 (*b*)	3 (*c*)	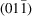	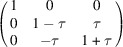	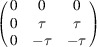	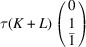
2 (*b*)	3 (*c*)	(011)	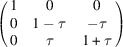	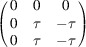	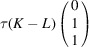

**Table 3:** The definitions of ferroelastic trigonal domains (organized in the same way as Table 1 ▸)

Domain variant	1	2	3	4
[*G*]_*m*_	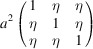	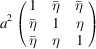	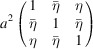	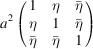
Unique axis	[111]	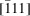	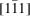	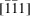

**Table 4:** The same as Table 2 ▸ but for the rhombohedral domains

*m*	*n*	Plane	[*S*]	[*S* ^*^] − [*I*]	Δ**B**
1	2	(011)	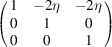	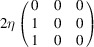	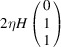
1	2	(100)	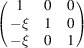	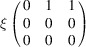	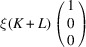
1	3	(101)	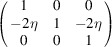	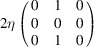	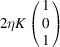
1	3	(010)	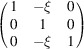	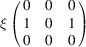	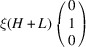
1	4	(110)	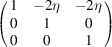	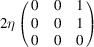	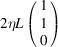
1	4	(001)	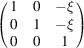	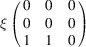	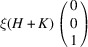
2	3	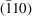	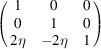	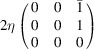	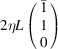
2	3	(001)	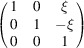	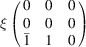	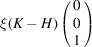
2	4	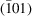	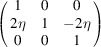	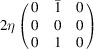	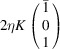
2	4	(010)	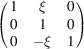	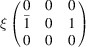	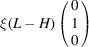
3	4	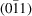	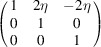	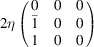	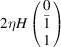
3	4	(100)	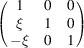	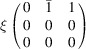	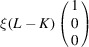

**Table 5:** A summary of the individual sub-peaks marked in the 102 reciprocal-space map The top row gives the peak numbers, the second row gives the corresponding length of the reciprocal-space vector [ 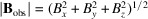 ], the third row gives the best matching calculated length of the reciprocal-lattice vector [ 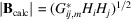 ], the fourth row gives the domain number(s) *m* for which this matching is achieved. The three bottom rows give the reciprocal-lattice coordinates ( 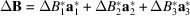 ) of all the peaks with respect to the peak centre of gravity.

Sub-peak No.	1	2	3	4	5	6
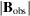 (Å^−1^)	0.5645	0.5648	0.5595	0.5598	0.5635	0.5645
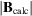 (Å^−1^)	0.5646	0.5646	0.5598	0.5598	0.5634	0.5646
Domain assignments	2	2	3	3	1	2
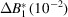	1.4	0.2	1.3	1.4	−0.9	1.4
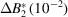	−1.4	−0.7	−0.6	0.8	0.4	1.7
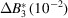	−0.1	0.6	−2.3	−2.4	0.6	−0.1

**Table 6:** Identification of a coherent twin relationship using 102 families of Bragg peaks The first two columns show domain numbers. The third and fourth columns indicate the expected separation between the peaks in the analytical and numerical form correspondingly. The last column shows the best matching (when such matching is found) separation between the Bragg peaks and their numbers according to Table 5 ▸.

*m*	*n*	Δ**B** (equation)	Δ**B** (calculated), 10^−2^	Δ**B** (measured), 10^−2^	Sub-peaks pair [according to the numbering in Figs. 6 ▸(*a*)–6(*c*)]	
1(*a*)	2(*b*)	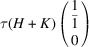	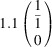	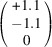	5	2
2(*b*)	3(*c*)	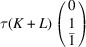	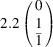	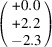	1	4
2(*b*)	3(*c*)	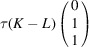	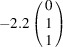	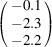	6	3

**Table 7:** The coordinates of individual sub-peaks (in the reciprocal-lattice units) The error bar of 0.001 is assumed for each number in the table.

Sub-peak No.	1	2	3	4
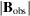 (Å^−1^)	1.1162	1.1138	1.1105	1.1102
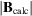 (Å^−1^)	1.1162	1.1139	1.1104	1.1104
Domain assignments	3	2	1	1
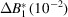	−0.5	−0.4	−0.6	−2.1
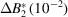	1.7	1.7	0.7	1.6
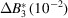	1.6	0.4	−0.6	−0.8

**Table 8:** Identification of a coherent twin relationship in the 124 reciprocal-space maps of a rhombohedral PbZr_0.75_Ti_0.25_O_3_ crystal The first two columns show domain numbers (and names). The third and fourth columns indicate the expected separation between the peaks in the analytical and numerical form correspondingly. The last column shows the best matching separation between the Bragg peaks and their numbers according to Table 5 ▸.

*m*	*n*	Δ**B** (equation)	Δ**B** (calculated), 10^−2^	Δ**B** (measured), 10^−2^	Sub-peaks pairs [according to the numbering in Figs. 8 ▸(*a*)–8(*c*)]	
1	2	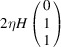	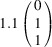	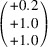	3	2
2	3	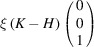	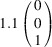	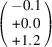	2	1
1	3	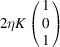	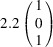	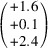	4	1

## Appendix A Derivation of equation (4)

This appendix proves equation (4) for the relationship between the metric tensors of domains *m* and *n*, related by the twinning matrix [*T*]. To do this let us introduce the matrix [*U*], describing the distortion of the crystallographic coordinate system **a**_*i*0_ during the phase transition. The columns of this matrix represent the coordinates of the matrix **a**_1*m*_, **a**_2*m*_, **a**_3*m*_ relative to the vectors **a**_10_, **a**_20_, **a**_30_:

Here, we do not consider any orientation relationship between the paraelastic and ferroelastic phase (even if such exist); therefore the elements of [*U*] are generally unknown. However, according to equation (2), the following relationship between [*G*]_0_ and [*G*]_*m*_ applies:

We assume that domain *n* has the same orientation relationship as domain *m* just with respect to the coordinate system  [see equation (3)]. This means that the lattice of the domain *n* can be described by the basis vectors  such that

Considering the definition of the twinning matrix [*T*] (3) we can rewrite (60) in the form

We will now transform the basis vectors  to **a**_*in*_ so that **a**_*in*_ are nearly parallel to the vectors **a**_*im*_. For that, we will apply the transformation matrix [*T*] that maps  back into **a**_*i*0_:

We can now apply equation (59) to calculate [*G*]_*n*_ just replacing [*U*] by [*T*]^−1^[*U*][*T*] there:

Considering that [*T*] (as well as [*T*]^−1^) is the symmetry operation for the parent-phase lattice, we can immediately say that [*G*_0_] = [[*T*]^−1^]^T^[*G*_0_][*T*]^−1^. This immediately leads to
