# Supplementary material for: Identification of a coherent twin relationship from high-resolution reciprocal-space maps
Source: Acta Crystallogr A Found Adv. 2022 Apr 28;78(Pt 3):158–71. doi: 10.1107/S2053273322002534 (PMC9062825; doi:10.1107/S2053273322002534)
Supplement: Supplementary file 1 [file a-78-00158-sup1.pdf]

Acta Cryst A

FOUNDATIONS
ADVANCES

Volume 78 (2022)

Supporting information for article:

Identification of a coherent twin relationship from high-resolution reciprocal-space maps

Semën Gorfman, David Spirito, Guanjie Zhang, Carsten Detlefs and Nan Zhang

S1. Recognition of peaks in three-dimensional reciprocal space maps of $BaTiO_3$.

This supporting information demonstrates the assignments of sub-peaks to domains in the reciprocal space maps of tetragonal $BaTiO_3$ crystal. The organization of figures and tables is exactly as in §7. Figures S1, S2, S3 (identical to Figure 6) show $I_z(H_x, H_y)$, $I_y(H_x, H_z)$ and $I_x(H_y, H_z)$ projections of diffraction intensity distribution around 002, 222 and 013 families of Bragg peaks correspondingly. The individual sub-peaks are explicitly marked. Tables S1, S3 and S5 (identical to Table 5) summarize the positions of individual sub-peaks in the corresponding figures. Table S2, S4, S6 (identical to Table 6) illustrate the assignment of the peaks / peaks pairs to the matched domain pairs.

S1.1. Example 1: splitting of 002 reflection / $BaTiO_3$.

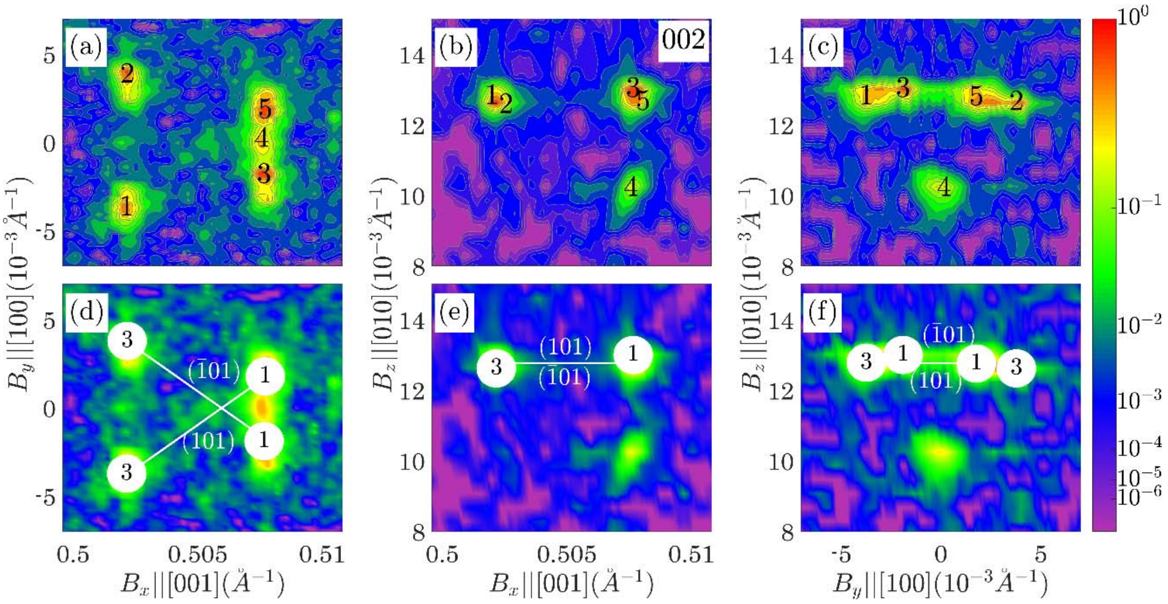

Figure S1 The same as Figure 6 but for the case of intensity distribution around 002 reflection from multi-domain $BaTiO_3$ crystal.

Table S1 The summary of the individually marked sub-peaks in the 002 reciprocal space map. The top row gives the peaks numbers (per their marking in Figure S1), the second row gives the corresponding length of the reciprocal space vector ($|\boldsymbol{B}_{obs}| = \sqrt{B_x^2 + B_y^2 + B_z^2}$), the third row gives the best matching calculated length of the reciprocal lattice vector ($|\boldsymbol{B}_{calc}| = \sqrt{G^*_{mij} H_i H_j}$), the forth row gives the domain number(s) m for which this matching is achieved. Three bottom rows give the reciprocal lattice coordinates ($\Delta\boldsymbol{B} = \Delta B_1 \boldsymbol{a}_1^* + \Delta B_2 \boldsymbol{a}_2^* + \Delta B_3 \boldsymbol{a}_3^*$) of all the peaks relative to the peak center of gravity

Sub-peak number		1	2	3	4	5
$\lvert\boldsymbol{B}_{obs}\rvert$, Å^{-1}		0.5025	0.5025	0.5080	0.5075	0.5080
$\lvert\boldsymbol{B}_{calc}\rvert$, Å^{-1}		0.5025	0.5025	0.5079	0.5079	0.5079
Domain assignments		$3(c)$	$3(c)$	$1(a), 2(b)$	$1(a), 2(b)$	$1(a),2(b)$
$\Delta\boldsymbol{B}$	ΔB_1^* (10^{-2})	-1.6	1.4	-0.9	0.0	0.6
	ΔB_2^* (10^{-2})	-0.3	-0.4	-0.2	-1.3	-0.3
	ΔB_3^* (10^{-2})	-1.3	-1.3	0.9	0.8	0.9

Table S2 **Identification of coherent twin relationship present among 002 families of Bragg peaks.** The first two columns show domain numbers and names (per definitions in Figure 3). The third and four's column indicate the expected separation between the peaks in the analytical and numerical form correspondingly. The fifth column shows the best matching (when reasonable match is found) separation between the Bragg peaks and their numbers according to the Table S1.

m	n	$\Delta\boldsymbol{B}$ (equation)	$\Delta\boldsymbol{B}$ (calculated), 10^{-2}	$\Delta\boldsymbol{B}$ (measured) 10^{-2}	Sub peaks pair (According the numbering in Figure S1)	
$1(a)$	$3(c)$	$\tau(H+L)\begin{pmatrix}1\\0\\\bar{1}\end{pmatrix}$	$+2.2\begin{pmatrix}1\\0\\\bar{1}\end{pmatrix}$	$\begin{pmatrix}+2.3\\-0.2\\-2.2\end{pmatrix}$	3	2
$1(a)$	$3(c)$	$\tau(H-L)\begin{pmatrix}1\\0\\1\end{pmatrix}$	$-2.2\begin{pmatrix}1\\0\\1\end{pmatrix}$	$\begin{pmatrix}-2.2\\+0.0\\-2.2\end{pmatrix}$	5	1

Accordingly, the following assignment can be made.

- The sub-peaks 3 and 2 are assigned to the tetragonal domains $1(a)$ and $3(c)$. These domains meet along $(10\bar{1})$ oriented domain wall.
- The sub-peaks 5 and 1 are assigned to the tetragonal domains $1(a)$ and $3(c)$. These domains meet along (101) oriented domain wall.

Note, that no unambiguous assignment could be done for the sub-peak 4. If such assignment were made based on the length of the reciprocal lattice vector alone then this sub-peak would be associated with either domain $1(a)$ or domain $2(b)$.

S1.2. Example 2: Splitting of 222 reflection / $BaTiO_3$

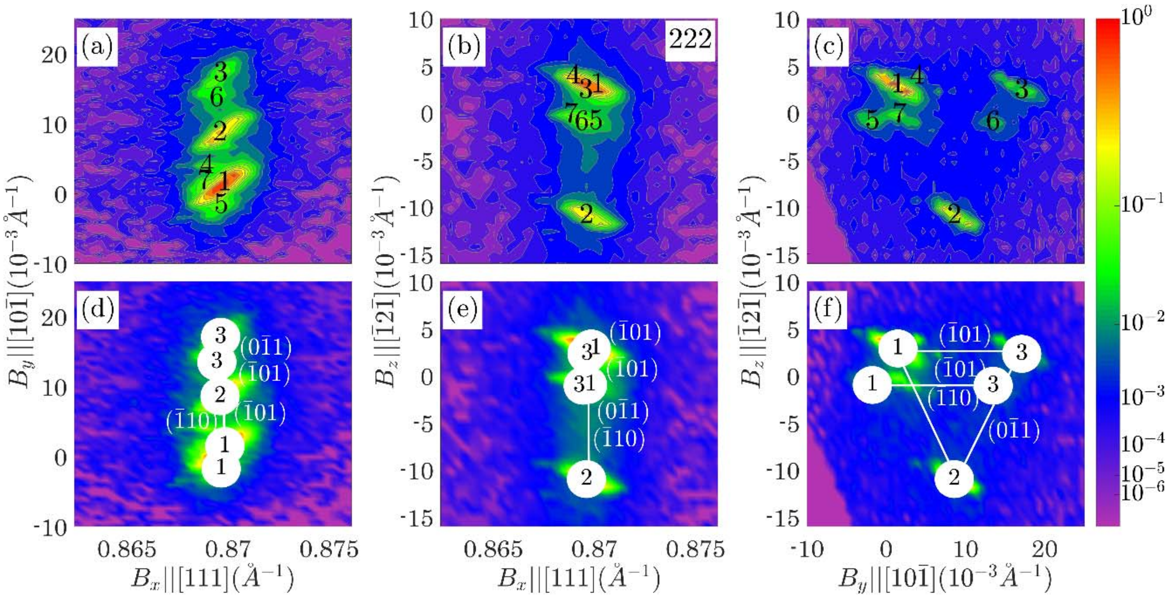

Figure S2 The same as Figure S1 but for the case of 222 reflection from multi-domain $BaTiO_3$ crystal.

Table S3 The same as Table S1 but for the case of 222 reflection from $BaTiO_3$ crystal.

	Sub-peak number	1	2	3	4	5	6	7
	$\|\boldsymbol{B}_{obs}\|$, Å^{-1}	0.8698	0.8695	0.8700	0.8690	0.8698	0.8695	0.8697
	$\|\boldsymbol{B}_{calc}\|$, Å^{-1}	0.8695	0.8695	0.8695	0.8695	0.8695	0.8695	0.8695
	Domain assignments	all	all	all	all	all	all	All
	ΔB_1^* (10^{-2})	-1.6	2.6	2.9	-1.2	-1.9	2.4	-1.2
$\Delta\boldsymbol{B}$	ΔB_2^* (10^{-2})	1.4	-3.2	1.1	1.4	0.1	0.0	0.1
	ΔB_3^* (10^{-2})	0.2	0.5	-4.0	-0.8	1.8	-2.6	0.5

Table S4 The same as Table S2 but for the case of 222 reflection from $BaTiO_3$ crystal.

m	n	$\Delta\boldsymbol{B}$ (equation)	$\Delta\boldsymbol{B}$ (calculated), 10^{-2}	$\Delta\boldsymbol{B}$ (measured) 10^{-2}	Sub peaks pair, m and n (According the numbering in Figure S2)	
$1(a)$	$2(b)$	$\tau(H+K)\begin{pmatrix}1\\\bar{1}\\0\end{pmatrix}$	$+4.3\begin{pmatrix}1\\\bar{1}\\0\end{pmatrix}$	$\begin{pmatrix}+4.2\\-4.6\\+0.3\end{pmatrix}$	1	2

$1(a)$	$3(c)$	$\tau(H+L)\begin{pmatrix}1\\0\\\bar{1}\end{pmatrix}$	$+4.3\begin{pmatrix}1\\0\\\bar{1}\end{pmatrix}$	$\begin{pmatrix}+4.3\\-0.1\\-4.4\end{pmatrix}$	5	6
$1(a)$	$3(c)$	$\tau(H+L)\begin{pmatrix}1\\0\\\bar{1}\end{pmatrix}$	$+4.3\begin{pmatrix}1\\0\\\bar{1}\end{pmatrix}$	$\begin{pmatrix}+4.5\\-0.3\\-4.2\end{pmatrix}$	1	3
$2(b)$	$3(c)$	$\tau(K+L)\begin{pmatrix}0\\1\\\bar{1}\end{pmatrix}$	$+4.3\begin{pmatrix}0\\1\\\bar{1}\end{pmatrix}$	$\begin{pmatrix}+0.3\\+4.3\\-4.5\end{pmatrix}$	2	3

Accordingly, the following assignment can be made:

- The sub-peaks 1 and 2 are assigned to the tetragonal domains $1(a)$ and $2(b)$. These domains are matched along $(1\bar{1}0)$ oriented domain wall.
- The sub-peaks 1 and 3 are assigned to the tetragonal domains $1(a)$ and $3(c)$. These domains meet along $(10\bar{1})$ domain wall.
- The sub-peaks 5 and 6 can be assigned to the tetragonal domains $1(a)$ and $3(c)$. These domains meet along $(10\bar{1})$ domain wall.
- The sub-peaks 2 and 3 are assigned to the tetragonal domains $2(b)$ and $3(c)$. These domains meet along $(01\bar{1})$ domain wall.

S1.3. Example 3: Splitting of 013 reflection / $BaTiO_3$

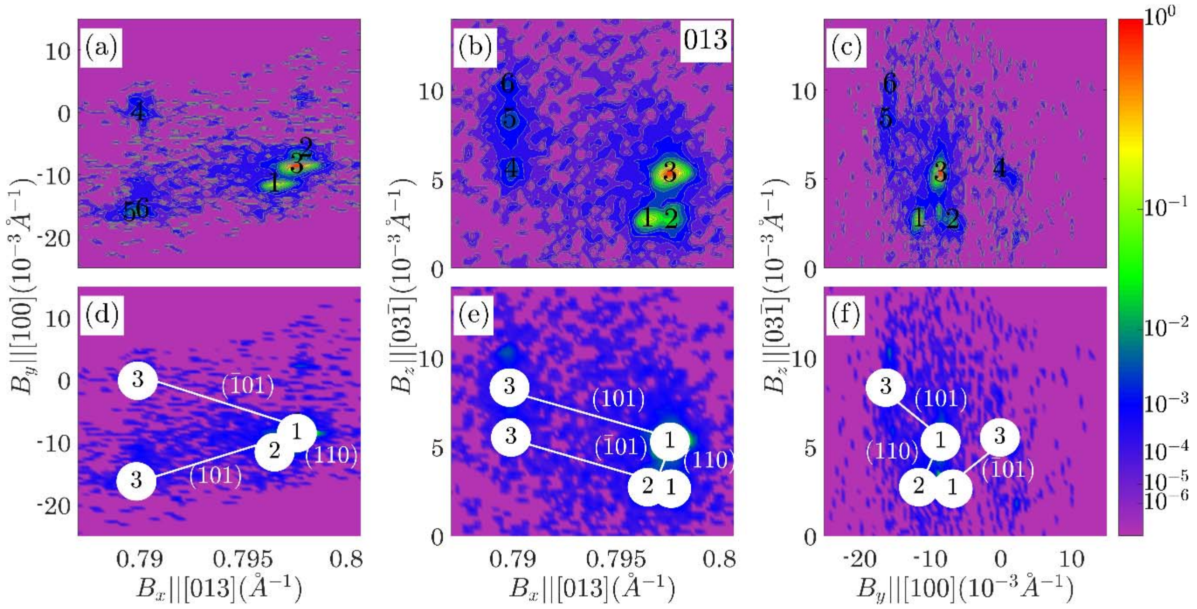

Figure S3 The same as in the Figure S1 but for the case of 013 reflection from multi-domain $BaTiO_3$ crystal.

Table S5 The same as Table S1 but for the case of 013 reflection from multi-domain $BaTiO_3$ crystal.

Sub-peak number		1	2	3	4	5	6
$\lvert \boldsymbol{B}_{obs} \rvert, Å^{-1}$		0.7965	0.7977	0.7975	0.7900	0.7900	0.7900
$\lvert \boldsymbol{B}_{calc} \rvert, Å^{-1}$		0.7969	0.7977	0.7977	0.7900	0.7900	0.7900
Domain assignments		2	1	1	3	3	3
	$\Delta B_1^*\ (10^{-2})$	-1.0	0.9	0.2	3.6	-2.9	-2.7
$\Delta\boldsymbol{B}$	$\Delta B_2^*\ (10^{-2})$	-0.9	-0.8	0.1	-0.7	0.3	1.0
	$\Delta B_3^*\ (10^{-2})$	0.0	0.5	0.1	-2.8	-3.2	-3.4

Table S6 The same as Table S2 but for the case of 013 reflection from $BaTiO_3$ crystal.

m	n	$\Delta\boldsymbol{B}$ (equation)	$\Delta\boldsymbol{B}$ (calculated), 10^{-2}	$\Delta\boldsymbol{B}$ (measured) 10^{-2}	Sub peaks pair, m and n (According the numbering in Figure S3)	
$1(a)$	$2(b)$	$\tau(H-K)\begin{pmatrix}1\\1\\0\end{pmatrix}$	$-1.1\begin{pmatrix}1\\1\\0\end{pmatrix}$	$\begin{pmatrix}-1.2\\-1.0\\-0.1\end{pmatrix}$	3	1
$1(a)$	$3(c)$	$\tau(H+L)\begin{pmatrix}1\\0\\\bar{1}\end{pmatrix}$	$3.2\begin{pmatrix}1\\0\\\bar{1}\end{pmatrix}$	$\begin{pmatrix}+2.7\\+0.1\\-3.3\end{pmatrix}$	2	4
$1(a)$	$3(c)$	$\tau(H-L)\begin{pmatrix}1\\0\\1\end{pmatrix}$	$-3.2\begin{pmatrix}1\\0\\1\end{pmatrix}$	$\begin{pmatrix}-3.1\\+0.2\\-3.3\end{pmatrix}$	3	5

Accordingly, the following assignment can be made:

- The sub-peaks 3 and 1 are assigned to the tetragonal domains $1(a)$ and $2(b)$. These domains meet along (110) domain wall.
- The sub-peaks 2 and 4 can be assigned to the tetragonal domains $1(a)$ and $3(c)$ and these peaks are matched along $(10\bar{1})$-oriented domain wall.
- The sub-peaks 3 and 5 can be assigned to the tetragonal domains $1(a)$ and $3(c)$ and these peaks are matched along (101) domain wall.

S2. Recognition of peaks in three-dimensional reciprocal space maps of $PbZr_{0.75}Ti_{0.25}O_3$.

This supporting information shows several examples of assignments of sub-peaks to domains in rhombohedral PZT crystal. The figures and tables are organized exactly as in §7. Specifically Figures S7, S9, S11 (identical to Figure 6) show $I_2(H_x, H_y)$, $I_2(H_x, H_z)$ and $I_2(H_y, H_z)$ projections of diffraction intensity distribution around 013, $\bar{1}33$ and 233 Bragg reflections correspondingly where individual sub-peaks are explicitly marked. Tables S7, S9 and S11 (identical to Table 5) summarize the

positions of individual sub-peaks in the corresponding figures. Table S8, S10, S12 (identical to Table 6) illustrate the assignment of the peaks / peaks pairs to the matched domain pairs. These connections are illustrated in Figure S8, S10, S12.

S2.1. Example 1: Splitting of 013 reflection / PZT

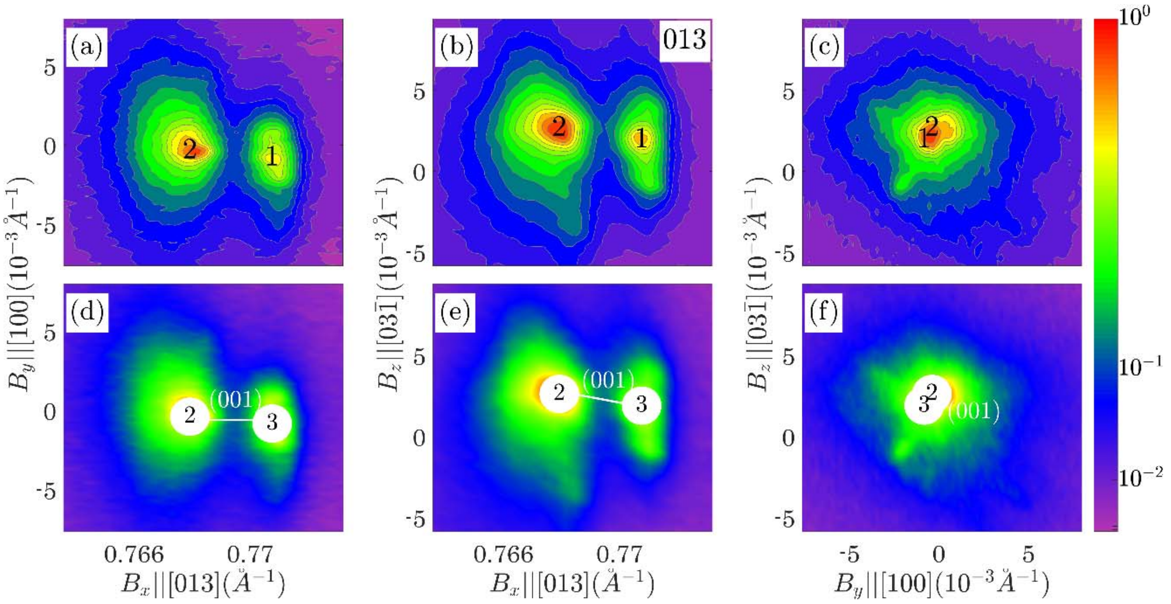

Figure S4 The same as Figure S1 but for the case of 013 reflections from multi-domain PZT crystal.

Table S7 The same as Table S1 but for the case of 013 reflection from multi-domain PZT crystal.

	Sub-peak number	1	2
	$\|\boldsymbol{B}_{obs}\|$, Å^{-1}	0.7708	0.7680
	$\|\boldsymbol{B}_{calc}\|$, Å^{-1}	0.7708	0.7682
	Domain assignments	3 or 4	1 or 2
	ΔB_1^* (10^{-2})	-0.2	-0.1
$\Delta\boldsymbol{B}$	ΔB_2^* (10^{-2})	0.5	0.4
	ΔB_3^* (10^{-2})	1.0	-0.1

Table S8 The same as Table S2 but for the case of 013 reflections from multi-domain PZT crystal.

m	n	$\Delta\boldsymbol{B}$ (equation)	$\Delta\boldsymbol{B}$ (calculated), 10^{-2}	$\Delta\boldsymbol{B}$ (measured) 10^{-2}	Sub peaks pair, m and n (According the numbering in Figure S4)	
2	3	$\xi(K-H)\begin{pmatrix}0\\0\\1\end{pmatrix}$	$1.1\begin{pmatrix}0\\0\\1\end{pmatrix}$	$\begin{pmatrix}-0.1\\+0.1\\+1.1\end{pmatrix}$	2	1
1	4	$\xi(H+K)\begin{pmatrix}0\\0\\1\end{pmatrix}$	$1.1\begin{pmatrix}0\\0\\1\end{pmatrix}$	$\begin{pmatrix}-0.1\\+0.1\\+1.1\end{pmatrix}$	2	1

This example demonstrate the case when ambiguous assignment of sub-peaks to domains is not possible. Any of the two following assignment can be suggested.

- The sub-peaks 2 and 1 are assigned to the rhombohedral domains 2 and 3. These domains meet (001) domain wall.
- Alternatively, the sub-peaks 2 and 1 are assigned to the rhombohedral domains 1 and 4, meeting along (001)-oriented domain wall too.

S2.2. Example 2: Splitting of 113 reflection / PZT.

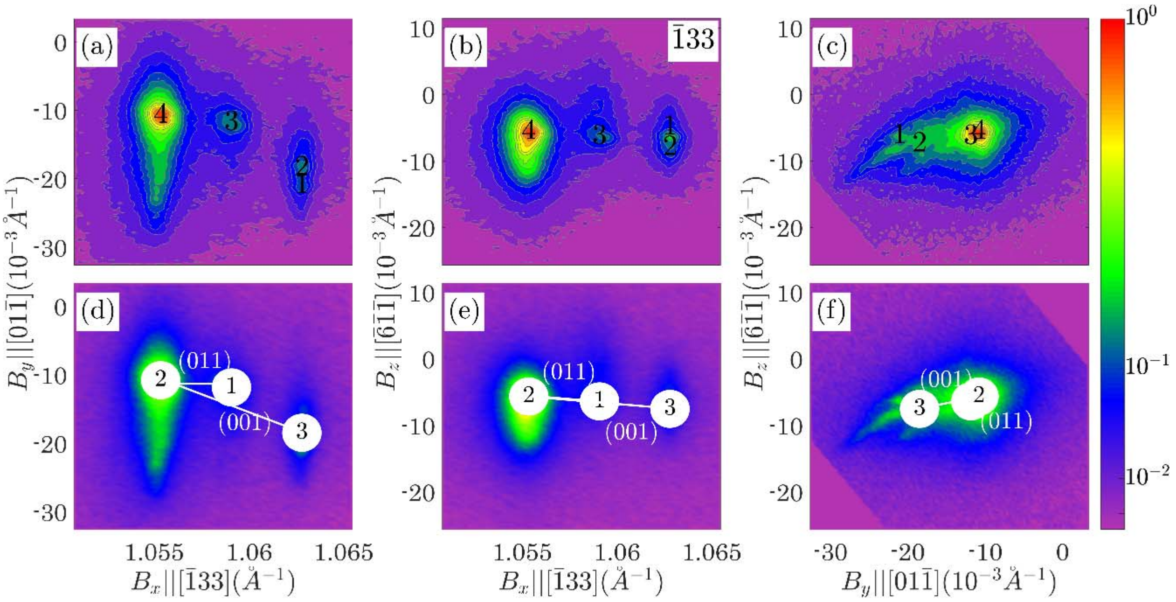

Figure S5 The same as Figure S1 but for the case of $\bar{1}33$ reflections from multi-domain PZT crystal.

Table S9 The same as Table S2 but for the case of $\bar{1}33$ family of reflections from multi-domain PZT crystal

	Sub-peak number	1	2	3	4
	$\lvert \boldsymbol{B}_{obs} \rvert$, Å^{-1}	1.0630	1.0630	1.0590	1.0553
	$\lvert \boldsymbol{B}_{calc} \rvert$, Å^{-1}	1.0627	1.0627	1.0590	1.0553
	Domain assignments	3,4	3,4	1	2
	ΔB_1^* (10^{-2})	-0.7	-0.2	-0.2	-0.2
$\Delta\boldsymbol{B}$	ΔB_2^* (10^{-2})	-0.4	0.5	1.3	0.5
	ΔB_3^* (10^{-2})	3.8	3.2	0.1	-1.3

Table S10 The same as Table S2 but for the case of $\bar{1}13$ family of reflections of rhombohedral PZT crystal

m	n	$\Delta\boldsymbol{B}$ (equation)	$\Delta\boldsymbol{B}$ (calculated), 10^{-2}	$\Delta\boldsymbol{B}$ (measured) 10^{-2}	Sub peaks pair (According the numbering in Figure S5)	
1	2	$2\eta H\begin{pmatrix}0\\1\\1\end{pmatrix}$	$-1.1\begin{pmatrix}0\\1\\1\end{pmatrix}$	$\begin{pmatrix}+0.0\\-0.8\\-1.4\end{pmatrix}$	3	4

2	3	$\xi(K-H)\begin{pmatrix}0\\0\\1\end{pmatrix}$	$+4.4\begin{pmatrix}0\\0\\1\end{pmatrix}$	$\begin{pmatrix}+0.0\\+0.0\\+4.5\end{pmatrix}$	4	2

According to Table S10 and Figure 5 the following assignment can be made:

- The sub-peaks 3 and 4 are assigned to the rhombohedral domains 1 and 2. These domains meet along (011) domain wall.
- The sub-peaks 4 and 2 can be assigned to the tetragonal domains 2 and 3. These domains meet along (001) domain wall.

S2.3. Example 3: Splitting of 233 reflection / PZT

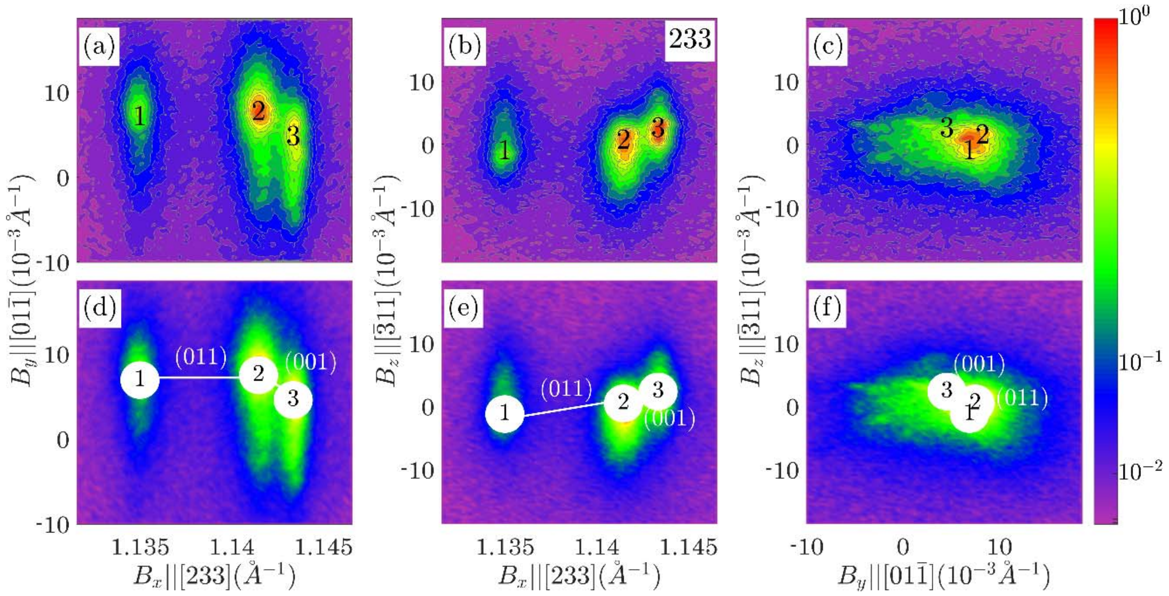

Figure S6 The same as Figure S1 but for the case of 233 reflections from PZT crystal.

Table S11 The same as Table S1 but for the case of 233 reflection of multi-domain PZT crystal

Sub-peak number		1	2	3
$\lvert \boldsymbol{B}_{obs}\rvert$, $Å^{-1}$		1.1350	1.1415	1.1433
$\lvert \boldsymbol{B}_{calc}\rvert$, $Å^{-1}$		1.1347	1.1415	1.1433
Domain assignments		1	2	3,4
	ΔB_1^* (10^{-2})	-1.4	-0.9	-1.3
$\Delta \boldsymbol{B}$	ΔB_2^* (10^{-2})	-1.7	0.3	0.2
	ΔB_3^* (10^{-2})	-2.1	-0.4	1.2

Table S12 The same as Table S2 but for the case of 233 family of reflections of rhombohedral PZT crystal

m	n	$\Delta\boldsymbol{B}$ (equation)	$\Delta\boldsymbol{B}$ (calculated), 10^{-2}	$\Delta\boldsymbol{B}$ (measured) 10^{-2}	Sub peaks pair (According the numbering in Figure S6)	
1	2	$2\eta H\begin{pmatrix}0\\1\\1\end{pmatrix}$	$2.2\begin{pmatrix}0\\1\\1\end{pmatrix}$	$\begin{pmatrix}+0.5\\+2.0\\+1.7\end{pmatrix}$	1	2
2	3	$\xi(K-H)\begin{pmatrix}0\\0\\1\end{pmatrix}$	$1.1\begin{pmatrix}0\\0\\1\end{pmatrix}$	$\begin{pmatrix}-0.4\\-0.1\\+1.6\end{pmatrix}$	2	3

According to Table S12 and Figure S6 the following assignment can be made:

- The sub-peaks 1 and 2 are assigned to the rhombohedral domains 1 and 2. These domains meet along (011) domain wall.
- The sub-peaks 2 and 3 can be assigned to the tetragonal domains 2 and 3. These domains meet along (001) domain wall.
